# Substantial improvement of toyocamycin production in *Streptomyces diastatochromogenes* by cumulative drug-resistance mutations

**DOI:** 10.1371/journal.pone.0203006

**Published:** 2018-08-30

**Authors:** Xu-Ping Shentu, Zhen-Yan Cao, Yin Xiao, Gu Tang, Kozo Ochi, Xiao-Ping Yu

**Affiliations:** 1 Zhejiang Provincial Key Laboratory of Biometrology and Inspection & Quarantine, College of Life Sciences, China Jiliang University, Hangzhou, China; 2 Department of Life Science, Hiroshima Institute of Technology, Hiroshima, Japan; Universite Paris-Sud, FRANCE

## Abstract

Toyocamycin is a member of the nucleoside antibiotic family and has been recognized as a promising fungicide for the control of plant diseases. However, low productivity of toyocamycin remains an important bottleneck in its industrial production. Therefore, dramatic improvements of strains for overproduction of toyocamycin are of great interest in applied microbiology research. In this study, we sequentially selected for mutations for multiple drug resistance to promote the overproduction of toyocamycin by *Streptomyces diastatochromogenes* 1628. The triple mutant strain, SD3145 (*str str par*), was obtained through sequential screenings. This strain showed an enhanced capacity to produce toyocamycin (1500 mg/L), 24-fold higher than the wild type in GYM liquid medium. This dramatic overproduction was attributed at least partially to the acquisition of an *rsmG* mutation and increased gene expression of *toyA*, which encodes a LuxR-family transcriptional regulator for toyocamycin biosynthesis. The expression of *toyF* and *toyG*, probably directly involved in toyocamycin biosynthesis, was also enhanced, contributing to toyocamycin overproduction. By addition of a small amount of scandium (ScCl_3_·6H_2_O), the mutant strain, SD3145, produced more toyocamycin (2664 mg/L) in TPM medium, which was the highest toyocamycin level produced in shake-flask fermentation by a streptomycete so far. We demonstrated that introduction of combined drug resistance mutations into *S*. *diastatochromogenes* 1628 resulted in an obvious increase in the toyocamycin production. The triple mutant strain, SD3145, generated in our study could be useful for improvement of industrial production of toyocamycin.

## Introduction

Members of the genus *Streptomyces* are the primary producers of numerous valuable secondary metabolites [1, 2]. It has been estimated that greater than 70% of the antibiotics developed for use both in medicine and agriculture are produced by *Streptomyces* [3]. The pyrrolopyrimidine, toyocamycin, is a member of the nucleoside antibiotic family and has been recognized as a promising fungicide for the control of plant diseases [4,5]. However, low productivity of toyocamycin remains an important bottleneck in its industrial production. Therefore, dramatic improvements of strains for overproduction of toyocamycin are of great interest.

‘Ribosome engineering’ has been developed to increase the expression of genes and antibiotic production in bacteria through the modulation of ribosomal components, including ribosomal proteins and rRNA[6,7]. This approach is based on the introduction of genetic mutations that confer resistance to ribosome-targeting drugs, including streptomycin, gentamicin, paromomycin, and others[7]. This approach holds several advantages including the ability to screen for spontaneous drug resistance mutations through a simple selection that can be carried out on drug-containing plates even in the case of mutations with extremely low frequency (e.g., <10^−10^) and the ability to select for mutations in the absence of known genetic information[8,9]. Furthermore, this method has been widely applied for strain improvement for antibiotic overproduction and even for novel antibiotic discovery[8,10,11].

In our previous work, a strain (No.1628) possessing the ability to synthesize toyocamycin was isolated and identified as *S*. *diastatochromogenes* 1628[4]. Recently, we have discovered that the acquisition of resistance to certain antibiotics, including paromomycin and streptomycin, improved the ability of *S*. *diastatochromogenes* to produce toyocamycin[11]. In the current study, we aimed to develop combinations of drug resistance mutations to further improve toyocamycin-producing strains. Here, we describe certain physiological aspects, as well as the expression levels of toyocamycin biosynthesis genes in the mutant strains generated in this study.

## Materials and methods

### Strains

The wild-type strain *S*. *diastatochromogenes* 1628 and its mutants used in this study are listed in Table 1. *S*. *diastatochromogenes* 1628 was deposited in the China general microbiological culture collection (CGMCC) and was assigned as accession number of CGMCC 2060. Spontaneous rifampicin-resistant (Rif^r^), streptomycin-resistant (Str^r^), gentamicin-resistant (Gen^r^), paromomycin-resistant (Par^r^), and fusidic acid-resistant (Fus^r^) mutants were obtained from colonies that grew within 5 to 10 days after spore suspensions were spread on GYM agar containing various concentrations of each respective antibiotic. All strains obtained were stored as spore suspensions at -80°C.

**Table 1:** Strains and summary of mutations on the *S*. *diastatochromogenes rpsL*, *rsmG* or *rpoB* gene resulting in amino acid exchange.

Strain	Genotype ^a^	Source^b^	Position in *rpsL* gene^c^	Position in *rsmG* gene^c^	Position in *rpoB* gene^c^	Amino acid substitution
1628	wild-type					
SD10	*str*	Low-level streptomycin-resistant mutant from 1628		177C→A		Cys59→stop codon
SD19	*str*	Low-level streptomycin-resistant mutant from 1628		448G→A		Gly163→Asp
SD26	*str*	Low-level streptomycin-resistant mutant from 1628		425C→B^d^		Frameshift
SD37	*str*	Low-level streptomycin-resistant mutant from 1628		26C→T		Pro9→Leu
				242T→G		Leu81→Arg
SD88	*rif*	Rifampicin-resistant mutant from 1628			1310C→T	Pro437→Leu
SD99	*par*	Paromomycin-resistant mutant from 1628	not found			
SD210	*str str*	High-level streptomycin-resistant mutant from SD10	not found	177C→A		Cys59→stop codon
SD228	*str str*	High-level streptomycin-resistant mutant from SD10	not found	177C→A		Cys59→stop codon
SD237	*str str*	High-level streptomycin-resistant mutant from SD10	not found	177C→A		Cys59→stop codon
SD252	*str gen*	Gentamicin-resistant mutant from SD10	not examined	177C→A		Cys59→stop codon
SD295	*rif str*	Low-level streptomycin-resistant mutant from SD88	not found		1310C→T	Pro437→Leu
SD3145	*str str par*	Paromomycin-resistant mutant from SD228	not found	177C→A		Cys59→stop codon
SD3176	*str str gen*	Gentamicin -resistant mutant from SD228	not found	177C→A		Cys59→stop codon
SD3196	*str str fus*	Fusidic acid -resistant mutant from SD228	not found	177C→A		Cys59→stop codon

^a^ Str: streptomycin, Rif: rifampicin, Par: paromomycin, Fus: fusidic acid, Gen: gentamicin.

^b^ All mutant strains isolated in this study were spontaneous antibiotic-resistant mutants.

^c^ Numbered in accordance with the numbering system for *S*. *coelicolor*.

^d^ B = AGGTGCACGTGGTGACCGC

### Media and growth conditions

GYM and 2XGYM media were described previously [12]. Toyocamycin producing medium (TPM) contained (per liter): soybean meal 40 g, bran 10 g, soluble starch 20 g, FeCl_2_ 1 g, CaCO_3_ 5 g, NH_4_NO_3_ 3 g, and KHSO_4_ 3 g. A spore suspension volume of 0.5 ml (approximately 1×10^7^ spores per ml) was inoculated into 50 ml of the above medium and was incubated at 28°C on a rotary shaker set to 200 rpm.

### Determination of MICs

The minimum inhibitory concentrations (MICs) were determined by spreading spore suspensions (~10^6^) onto GYM plates containing various antibiotic concentrations, followed by incubation at 28°C for the indicated time. The minimum drug concentration able to fully inhibit growth was defined as the MIC. The resistance levels were determined in a similar manner as the MIC.

### Analysis of toyocamycin

Toyocamycin production was determined using a Varian Prostar-240 HPLC (Prostar 240 Solvent Delivery Module, Prostar 335 PDA Detector, Prostar 410 Autosampler, Prostar Workstation, USA). A water-CH_3_OH gradient system was used, which ranged linearly from 5% to 100% CH_3_OH over the course of 30 min, and was then held for 10 min. The detection wavelength was 279 nm. The column (RP-C18 column) temperature was maintained at 30°C (250 mm×4.6 mm, 5 μm, XBridgeTM, Waters, USA)[11].

### Mutation analysis

The primers used to amplify candidate DNA fragments (*rsmG*, *rpoB* and *rpsL* genes) were designed as described previously [8,13]. PCR amplification was carried out using LA Taq (Takara), and the purified PCR products were sequenced by Shanghai Ruidi Biological Technology Co. The sequencing data were aligned using the Mega 5.0 program. To confirm the results, mutation analysis was conducted two to three times for each mutant strain.

### Transcriptional analysis by real-time qPCR

Total RNA was extracted and purified from cells grown on GYM medium for the indicated time using RNAiso Plus (TaKaRa) according to the manufacturer's instructions. One μg of each of the total RNA was used as a template for reverse transcription (RT), which was carried out using the PrimeScript RT reagent Kit with gDNA Eraser (Perfect Real Time) (TaKaRa). Realtime quantitative PCR (qRT-PCR) was carried out using an Applied Biosystems StepOnePlus Real-Time PCR System with SYBR^®^ *Premix Ex Taq*^TM^ (Tli RHaseH Plus) reagent (TaKaRa, Dalian, China). The gene primers used in qRT-PCR reactions are listed in S1 Table. Each reaction mixture was comprised of 7.5 μL of SYBR^®^ *Premix Ex Taq*^TM^ (2×), 1 μL of template, 0.3 μL of forward primer, 0.3 μL of reverse primer, 0.3 μL of ROX Reference Dye (50×), and 5.6 μL of RNase-free H_2_O. The acceptable qRT-PCR standard curve (0.9≤E≤1.0, R^2^≥0.99) for each gene examined in this study was optimized by altering the annealing temperature and time. For each gene, all PCR reactions were carried out in triplicate within a single plate, with *hrdB* of *S*. *diastatochromogenes* 1628 used as the reference gene. Quantification of relative gene expression was analyzed using the 2^-ΔΔCt^ method[14].

### Dry weight of biomass

Culture broth was filtered through filter paper and washed twice with reverse osmosis water in order to remove any residual medium remaining on the cell surface. The remaining cells were then dried in an oven at 55°C to a constant weight. Dry cell weights were determined by subtracting the weight of the filter paper from the weight of the filter paper plus the cells.

## Results

### Construction of combined resistant mutants

First, a single drug-resistant mutation was introduced into the wild-type strain 1628, as summarized in Table 2. A total of 218 spontaneous antibiotic-resistant mutants of strain 1628 were isolated from GYM agar plates containing rifampicin, streptomycin, or paromomycin at concentrations 2–10 fold higher than their respective MICs. Mutants that possessed enhanced toyocamycin production were detected at a high frequency (9% to 26%) among streptomycin-, paromomycin-, and rifampicin-resistant isolates. The highest productivity detected for each mutant strain ranged from 1.5 to 4.1 times of the wild-type production level. All of these drug resistance screenings were found to be effective in toyocamycin overproduction by *S*. *diastatochromogenes* 1628.

**Table 2:** Screening and toyocamycin productivity of drug-resistant mutants.

Strain	Toyocamycin produced (mg/L) ^a^	Toyocamycin titer/dry cell weight(mg/L/g)	MIC (mg/L) ^b^	Antibiotic concentration used for screening (mg /L)	Frequency (%) of mutants producing increased antibiotic^c^
1628	63	23.2	Streptomycin (5)	20	26 (20/78)
				50	21 (16/76)
			Rifampicin (1)	5	9 (3/34)
			Paromomycin (10)	20	20 (6/30)
SD10	134	29.8	Streptomycin (50)	1000	16(6/37)
			Fusidic acid (1)	3	12(3/25)
			Gentamicin (5)	8	14(9/64)
SD228	563	165	Paromomycin (10)	50	11(4/35)
			Gentamicin (3)	6	13(2/15)
			Fusidic acid (1)	3	15(4/26)
SD3145	1500	395			
SD3176	1250	318			
SD3195	1186	288			

^a^ Determined after 6 days of incubation at 28°C, using a 300-mL flask containing 50 mL of GYM medium.

^b^ Determined after 6 days of incubation on GYM agar medium at 28°C.

^c^ Mutants producing more antibiotic than starting strain. Numbers in parentheses show the number of mutants producing more antibiotic divided by the number of mutants tested.

Next, we created double mutant strains by generating spontaneous Str^r^, Fus^r^, and Gen^r^ mutants from the starting *str* mutant SD10 strain (Tables 1 and 2). The frequency of double mutants, which were demonstrated to produce increased amounts of toyocamycin, was found to be 12% to 16%, with the highest productivity ranging from 1.5 to 4.2 times of the SD10 strain. These findings indicated that all the combinations of single-resistance mutations resulted in the generation of double mutants (*str str*, *str gen*, and *str fus*), which were all effective to increase toyocamycin production.

**Fig 1:**
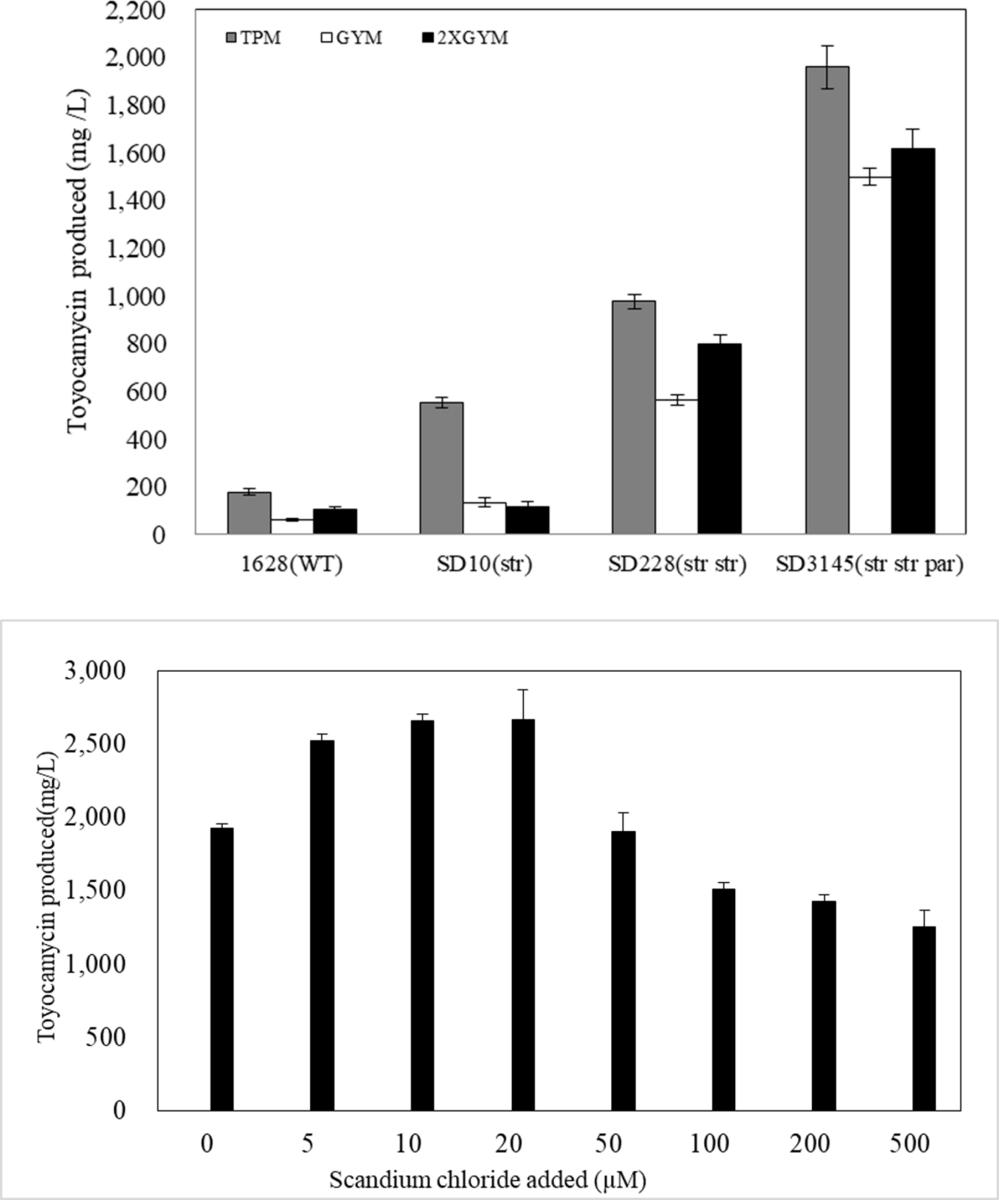
a Comparison of antibiotic production by the parent and mutant strains in three different liquid media (GYM, 2XGYM and toyocamycin production medium (TPM)). Antibiotic production was determined after a 6-day incubation at 28°C. The error bars indicate the standard deviations of the means of three or more samples. b Effects of scandium on toyocamycin production by the mutant SD3145 in TPM medium after a 6-day incubation at 28°C.

Finally, we created triple mutant strains by generating spontaneous *par*, *gen* and *fus* mutants in a *str str* double mutant strain (SD228) (Tables 1 and 2). The frequency of triple mutants to produce increased amounts of toyocamycin was found to be 11% to 15%, with the highest productivity found to be 1.86 times higher than that of the starting *str str* double-mutant strain. Thus, we demonstrated that the triple mutation (*par*, *gen* and *fus*) was effective in increasing toyocamycin productivity in strains containing double mutations. The toyocamycin titer of SD3145 with the highest productivity among the triple mutant strains reached 1500 mg/L (395 mg /L/g dry cell weight in yield), which was 24-fold of that produced by the wild type strain in GYM liquid medium (Fig 1A). A time course evaluation of toyocamycin production from wild-type and mutant strains was shown in S1 Fig. Thus, combinations of various drug resistance mutations had the ability to further enhance toyocamycin productivity.

### Mutation analyses of the mutants

Certain *rpsL*, *rsmG*, and *rpoB* mutations have been shown to have the ability to activate antibiotic production in *Streptomyces*[6,7]. Therefore, we sequenced and compared the *rpsL*, *rsmG* and *rpoB* genes between the mutants and wild-type strain. As summarized in Table 1, many of the low-level streptomycin-resistant mutants possessed an *rsmG* mutation, and these mutations in *rsmG* were effective at improving toyocamycin production in *S*. *diastatochromogenes* 1628. The mutation of the Cys residue at position 59 was often effective in other *Streptomyces*. The rifampicin-resistant mutant SD88 was found to possess a point mutation in the *rpoB* gene that generated an amino acid substitution from Pro437 to Leu. No mutation was identified in the *rpsL* gene of the high-level streptomycin-resistant and paromomycin-resistant mutants (Table 1), although the mutation site within the *rpsL* gene responsible for streptomycin or paromomycin resistance and antibiotic overproduction was identified in *S*. *coelicolor* A3(2) [15,16], implicating the presence of unknown mutations which are responsible for the enhanced toyocamycin production.

### Effects of different media on toyocamycin production

Toyocamycin production levels by wild-type and mutant strains were found to be medium-dependent (Fig 1A). We found that the mutant strain SD3145 produced 1959, 1500, and 1615 mg/L toyocamycin in TPM, GYM, and 2XGYM medium, respectively. These production levels were 11, 24, and 15 times of that produced by the wild-type strains (178, 63 and 105 mg/L), respectively. It was reported that the rare earth element, scandium (Sc), causes antibiotic overproduction when added at a low concentration (10–100 μM) to cultures of *S*. *coelicolor* A3(2) (actinorhodin producer), *S*. *antibioticus* (actinomycin producer), and *S*. *griseus* (streptomycin producer)[17]. Similarly, the addition of scandium (ScCl_3_·6H_2_O) markedly enhanced toyocamycin production by the mutant strain SD3145 when cells were grown in TPM medium for 6 days (Fig 1B). Scandium was effective at low concentrations (5–20 μM) and toyocamycin production was enhanced by 1.4-fold, reaching 2664 mg /L.

### Relative expression level of *toy* genes and cell growth in wild-type and mutant strains

**Fig 2:**
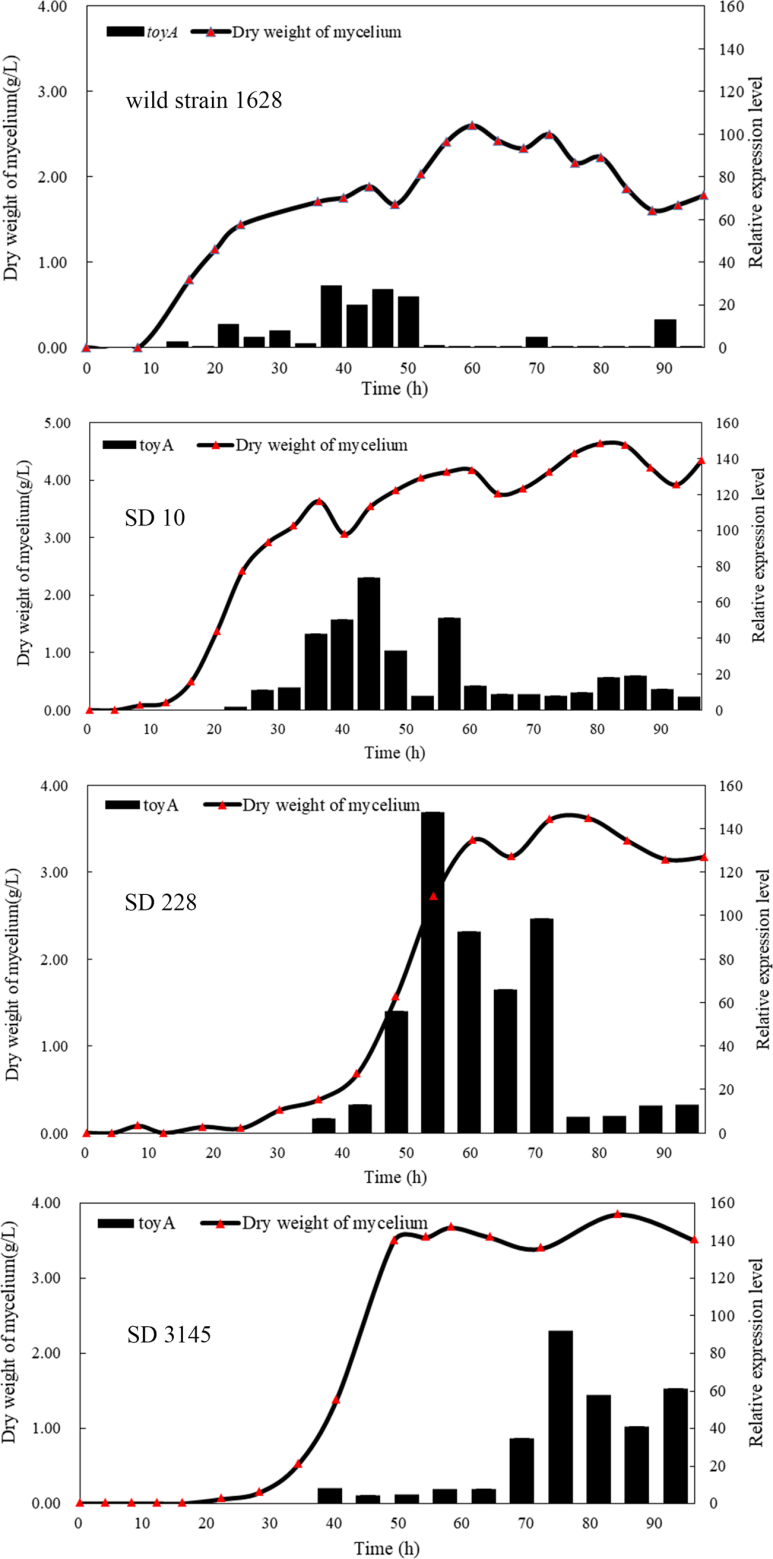
Relative expression level of *toyA* that acts as a LuxR transcriptional regulator in toyocamycin biosynthesis and the cell growth in the wild-type strain 1628 and mutant strains. Strains were grown in GYM medium, and total RNA preparation and real-time qPCR were performed as described in Materials and methods. The expression level at 68 h in the wild type strain was defined as 1.

Currently, the biosynthetic pathway of toyocamycin in *S*. *diastatochromogenes* has yet to be completely established. It was reported that *toyA* gene encodes a LuxR transcriptional regulator in the biosynthetic pathway of toyocamycin in *S*. *rimosus* (ATCC 14673) [18]. Certain mutations within the *rpoB*, *rpsL*, or *rsmG* genes have been demonstrated to dramatically enhance the expression of genes involved in the secondary metabolic biosynthesis of actinomycetes or *Bacillus subtilis*[13,19,20]. Thus, we studied the relative expression levels of the *toyA* gene using qRT-PCR, as well as cell growth of the wild-type and mutant strains (SD10, SD228 and SD3145). The profile of the changes in expression levels of the *toyA* gene is depicted in Fig 2. As expected, we observed remarkable increase in expression levels of *toyA* in the mutant strains SD10, SD228, and SD3145 relative to the wild-type strain at late growth phases (after 50 h). The relative expression levels of *toyA* reached its maximum value at 50 h and 75 h in the mutant strains SD228 and SD3145, respectively. These values were 4.2 and 2.5 times of that produced by wild-type strain (at 38 h), respectively. In the case of cell growth, the drug-resistant mutant strains grew more slowly compared to the wild-type strain. In contrast, the mycelium growth of the wild strain was observed to enter the logarithmic growth phase after 10 h. Overall, our findings regarding the mutant growth phenomenon were consistent with previous reports by Wang *et al*. [21]. The growth of the octuple mutant strain C8 of *S*. *coelicolor* was also very slow[21]. In general, it was found that drug resistance was obtained at the cost of growth fitness[22].

**Fig 3:**
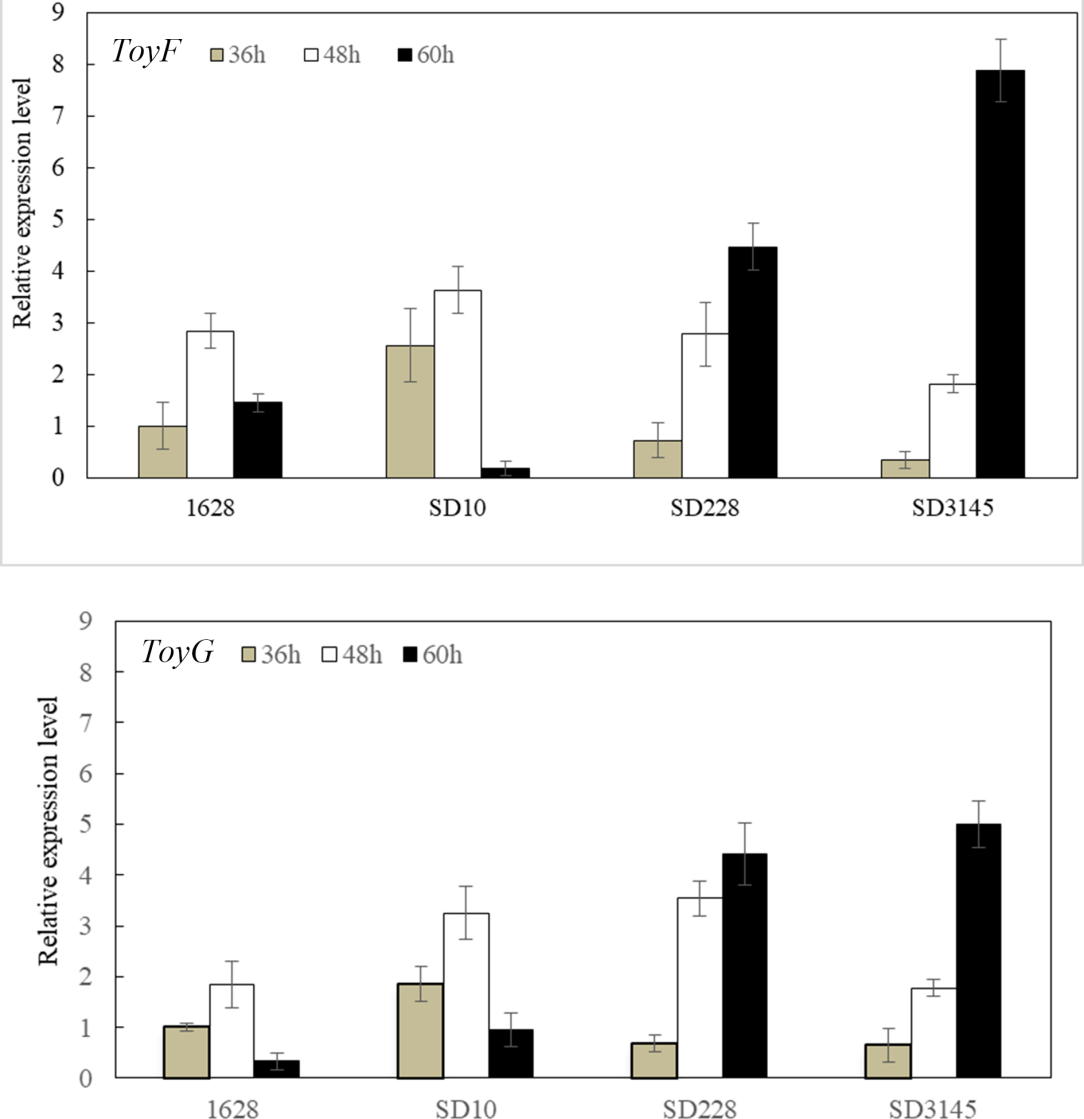
Relative expression levels of toyF and toyG involved in toyocamycin biosynthesis in the wild-type strain 1628 and mutant strains. Strains were grown in GYM medium, and total RNA preparation and real-time qPCR were performed as described in Materials and methods. The expression level at 36 h in the wild type strain was defined as 1.

Two fragments containing *toyF* and *toyG* were cloned previously in *S*. *diastatochromogenes* 1628. ToyF encodes an adenylosuccinate lyase and ToyG encodes an adenylosuccinate synthetase, both of which are probably involved in toyocamycin biosynthesis in *S*. *diastatochromogenes* 1628[18]. The relative expression levels of *toyF* and *toyG* genes in mutants and wild type strain were analyzed. As shown in Fig 3, the expression of *toyF* and *toyG* genes both in the wild and mutant strains changed at different time points (36, 48, and 60 h). There was significant increase in the expression of *toyF* and *toyG* in SD228 and SD3145 compared with control strain 1628 especially at time point of 60 h, likely due to the enhanced expression of transcriptional regulator *toyA* in the mutant strains. At 36 h, the expression of *toyF* and *toyG* was both lower in SD228 and SD3145 than in 1628 strain apparently due to their slow growth. Thus, the improvement of toyocamycin production can be attributed, at least partially, to the increase of the expression level of the genes directly involved in toyocamycin biosynthesis.

## Discussion

Ribosome engineering has been demonstrated to be an effective approach for improving antibiotic productivity in *S*. *diastatochromogenes* 1628[11]. Herein, we showed that combinations of various drug resistance mutations have the ability to further enhance antibiotic productivity. This has been previously demonstrated, for example, through the introduction of octuple drug-resistance mutations into *S*. *coelicolor* 1147 or triple drug-resistance mutations into *B*. *subtilis*. The octuple mutant C8 was found to produce large quantities (1.63 g/L) of antibiotic actinorhodin, which was 180-fold higher than that produced by the wild-type 1147 strain[21]. In our previous work, we showed that the Rif^r^(SD88), Str^r^(SD10, SD143), Gen(SD189)^r^, Par^r^(SD99), and Ery^r^(SD160) mutants were more antibiotic productive (toyocamycin, tetramycin P, tetrin B and Tetramycin A) than the wild type strain[11]. Here, we demonstrated the development of combined drug-resistant mutation approaches for further improvement of toyocamycin overproduction. We showed that the triple mutant SD3145 produced a higher yield (1959 mg/L) of the antibiotic toyocamycin, which was 11-fold greater than that produced by the wild-type 1628 strain. Our study proved that ribosome engineering represents a convenient, unlaborious, uncostly, and effective method for inducing antibiotic overproduction by bacteria. To enhance toyocamycin production by *S*. *diastatochromogenes* 1628, many methods such as intergeneric conjugation and adding a positive regulator for toyocamycin biosynthesis were used and the mutants 1628-VHB-23 and 1628-T62A produced 1.18-fold (165 mg/L) and 1.2-fold (181 mg/L) more of toyocamycin than the wild type, respectively. Apparently, ribosome engineering was more effective than the reported methods on toyocamycin overproduction [23–26].

The antibiotic streptomycin and rifampicin were frequently used as a screening drug by ribosome engineering technology and RNA polymerase engineering technology respectively due to their effectiveness on metabolite overproduction [8,27,28]. Therefore, we aimed to obtain spontaneous mutants through the introduction of specified *str* or *rif* mutations into *S*. *diastatochromogenes* 1628 in an effort to increase toyocamycin concentration. However, it remained difficult to obtain effective Rif^r^ mutants of *S*. *diastatochromogenes* 1628. As shown in Table 2, the frequency (%) of mutants that produce increased antibiotic levels was greater than 20% when streptomycin was used as the screening drug. In contrast, only 3 Rif^r^ mutants (i.e. 9% frequency) were found to produce enhanced toyocamycin. This may be explained by the fact that the frequency of spontaneous mutations in the *rpoB* gene of *S*. *diastatochromogenes* 1628 was very low (<10^−11^).

It was previously demonstrated that specific *rpsL* mutations that confer streptomycin or paromomycin resistance are effective in activating the antibiotic production in *Streptomyces* spp[13,29]. The mutations K88E and K88R (Lys→Glu and Lys→Arg at position 88, respectively) were most commonly identified as mutations associated with antibiotic overproduction. Currently, there are few reports regarding the paromomycin resistance mutation found in the *rpsL* gene. Wang *et al*. identified a novel paromomycin resistance-associated mutation in *rpsL* caused by the insertion of glycine residue at position 92 in *S*. *coelicolor* ribosomal protein S12[30]. This insertion mutation (GI92) was demonstrated to cause a 20-fold increase in the level of paromomycin resistance. However, no mutation was identified in the *rpsL* gene of the streptomycin- and paromomycin-resistant mutant SD3145, implicating the presence of unknown mutations which are responsible for the enhanced toyocamycin production. The *rpoB* mutations are often located at positions 1264C and 1327G, corresponding to the amino acid residues Leu422 and Ala443, respectively[8]. As expected, the rifampicin-resistant mutant SD88 was found to possess a point mutation in the *rpoB* gene, leading to a novel amino acid alteration from Pro437 to Leu.

In conclusion, we demonstrated that introduction of combined drug-resistant mutations into *S*. *diastatochromogenes* 1628 resulted in a 24-fold increase in the toyocamycin production in GYM medium. Although causal relationship between the increased toyocamycin production and each drug-resistance mutation was not demonstrated in the present work due to the lack of genetic system in this organism, the high frequency (9–26%) of high efficiency strains among the drug-resistant mutants shows a possible causality of these events. Scandium (ScCl_3_·6H_2_O) was effective for further enhancement of toyocamycin production. The triple mutant strain, SD3145, generated in our study could be useful for improvement of industrial production of toyocamycin. Previous work reported that the antibiotic productivity of the industrial *Streptomyces* spp. strain could be enhanced by 50- to 100-fold through the introduction of combined drug resistance-producing mutations [21,31]. We are currently planning to use the SD3145 strain for further development in a similar manner.

## Supporting information

S1 Fig The time course evaluation of toyocamycin production in GYM medium. (TIF)

S1 Table Primers used for qRT−PCR. (DOC)
