# Supplementary figures and images for: Substantial improvement of toyocamycin production in Streptomyces diastatochromogenes by cumulative drug-resistance mutations
Source: PLoS One. 2018 Aug 30;13(8):e0203006. doi: 10.1371/journal.pone.0203006 (PMC6117005; doi:10.1371/journal.pone.0203006)

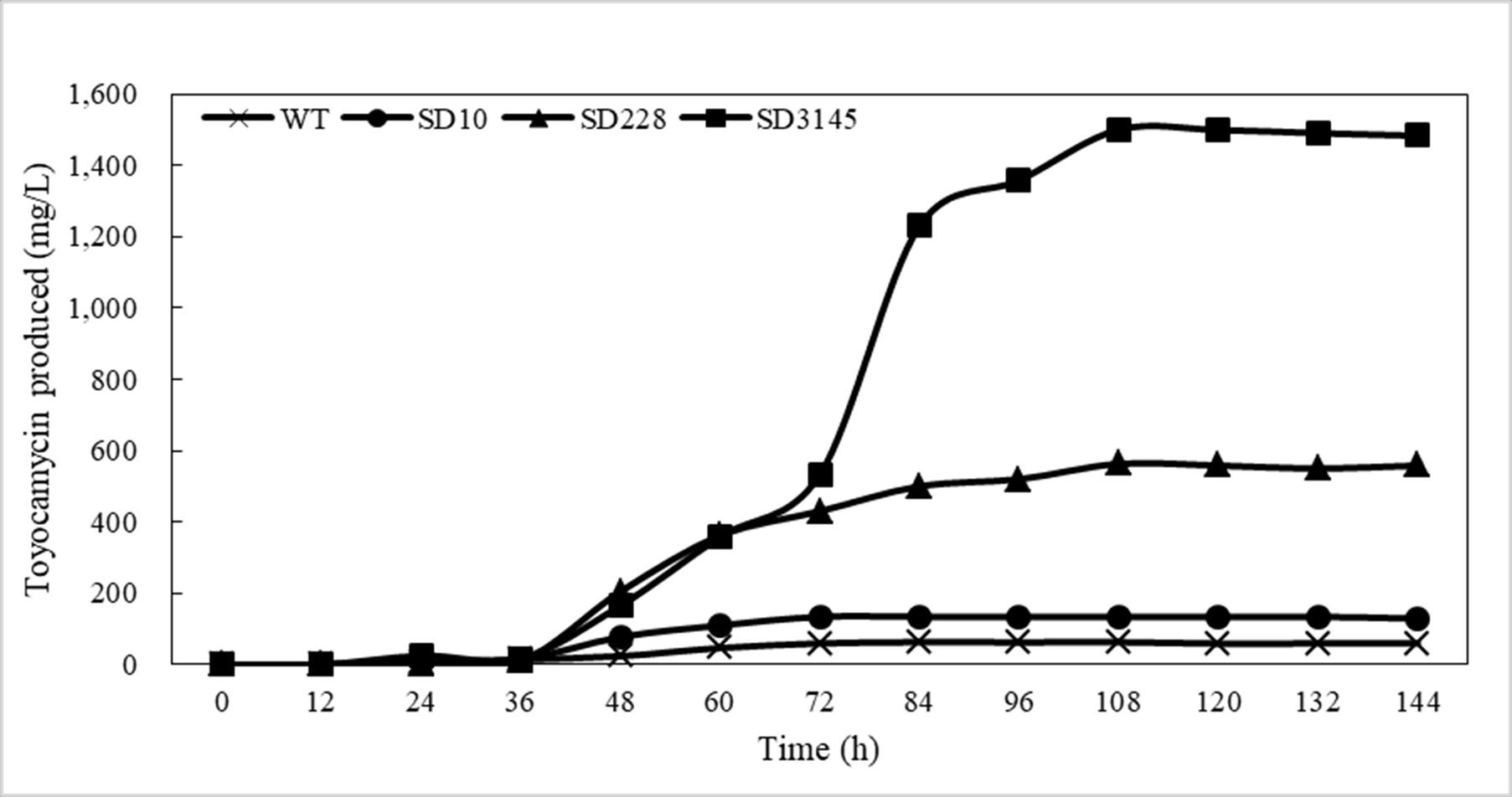

Supplement: S1 Fig — (TIF) [file pone.0203006.s001.tif]
