# Supplementary material for: Substantial improvement of toyocamycin production in Streptomyces diastatochromogenes by cumulative drug-resistance mutations
Source: PLoS One. 2018 Aug 30;13(8):e0203006. doi: 10.1371/journal.pone.0203006 (PMC6117005; doi:10.1371/journal.pone.0203006)
Supplement: S1 Table — (DOC) [file pone.0203006.s002.doc]

**S1 Table Primers used for qRT−PCR**

| **Gene name** | **Primer sequence(5’-3’)** |
| --- | --- |
| *toyA* F | CGACCTGTTGGTGTGCAT |
| *toyA* R | GGGCCATCAGCGTCAT |
| *toyF* F | GATGCCGCACAAGATGAAC |
| *toyF* R | GTCAGGAAGGTCTCCAGCAG |
| *toyG* F | AAGTACGCACTGCACCTCCT |
| *toyG* R | ACCTTGTCGGTGGTGATGTT |
| *hrdB* F | GGGCAACCTCGGTCTGATC |
| *hrdB* R | GAGAACTTGTAGCCCTTGGTGTAGT |
